# Supplementary material for: Large-scale DNA demethylation occurs in proliferating ovarian granulosa cells during mouse follicular development
Source: Commun Biol. 2021 Nov 25;4:1334. doi: 10.1038/s42003-021-02849-w (PMC8617273; doi:10.1038/s42003-021-02849-w)
Supplement: Supplementary file 2 — Supplementary Information [file 42003_2021_2849_MOESM2_ESM.pdf]

Supplemental Information

Large-scale DNA demethylation occurs in proliferating ovarian granulosa cells during mouse follicular development

Tomoko Kawai, JoAnne S. Richards and Masayuki Shimada

1. Supplementary Notes 1-6
2. Supplementary Table 1,2,3,4,5,6,7
3. Supplementary Figure 1,2,3,4,5,6,7,8

Supplementary Note 1: Experiment 1: The expression of genes involved in DNA methylation was analyzed in granulosa cells of secondary follicles or antral follicles before or after eCG injection, as shown in Supplementary Figure 7. The expression in the mRNA level was detected by real-time PCR analysis and is shown in Figure 1A, and the protein levels are shown by western blotting in Figures 1B and S6A. The localizations of DNMT1 and TET2 in follicles at each stage are shown in Figure 1C.

Supplementary Note 2: Experiment 2: To clarify the relationship between the level of DNMT1 and cell cycle stage in granulosa cells, granulosa cells collected from antral follicles in mouse ovaries at 12, 24 or 48 h after eCG injection were used for FACS analysis to detect DNMT1 and DNA levels (Figure 2).

Supplementary Note 3: Experiment 3: The low DNMT1 levels during the DNA replication process in the granulosa cells of preovulatory follicles suggested that DNA methylation copies might be limited during the DNA replication process. Therefore, we analyzed the DNA methylation status of granulosa cells during the follicle development process at the whole genome level. The analysis of genome-wide DNA methylation status in granulosa cells during follicular development is shown in Figure 3. The validation study of DNA methylation status by bisulfite sequence assay was performed in 3 selected genes (*Stk36*, *Trnau1ap* and *Lhcgr*) from the nine most highly demethylated genes (Figure 4A). Moreover, to examine the impacts of DNA demethylation on gene expression, open chromatin analysis by FAIRE-qPCR analysis (Figure 4B, Supplementary Figure 3A), chromatin immunoprecipitation assay of acetylated H3K27 (Figure 4C, Supplementary Figure 3B) and gene expression analysis by real-time PCR analysis (Figure 4D, Supplementary Figure 3C) were performed on the nine most highly demethylated genes.

Supplementary Note 4: Experiment 4: From the results of MeDIP-Seq analysis, the promoter regions of LH target genes (*Areg*, *Ereg*, *Btc*, *Star*, *Cyp11a1* and *Ptgs2*) were also significantly demethylated in granulosa cells by eCG stimulation (Figure 3). Therefore, to understand the mechanisms by which the LH target genes were expressed after hCG injection, FAIRE-qPCR analysis to detect the chromatin structure in the promoter region (Supplementary Figure 4A), ChIP assay using anti-H3K27 acetylated histone H3 (Supplementary Figure 4B) and real-time PCR analysis (Supplementary Figure 4C) were performed using granulosa cells just before hCG injection (at 48 hours after eCG injection) and granulosa cells at 4 or 8 h after hCG injection.

Supplementary Note 5: Experiment 5: The DNA methylation status in genome-wide promoter regions was dramatically changed in proliferated granulosa cells after eCG injection with

suppressing the induction of DNMT1 and increasing TET2. Therefore, to understand the relationship between cell proliferation and demethylation, the appearance pattern of demethylated CpG sites in each promoter region was analyzed by bisulfite sequencing (Figure 5A). Second, using an *in vitro* culture model to mimic the follicular development process, the effects of a cell cycle inhibitor (aphidicolin) on demethylation in promoter regions (Figure 5B) and gene expression (Figure 5C) were examined. The granulosa cells were cultured with FSH and testosterone (FSH+T) and/or aphidicolin for 48 h.

Supplementary Note 6: Experiment 6: Retinoic acid (RA) is one of the key inducers of *Lhcgr* expression in granulosa cells, and coculture with oocytes suppresses it. Therefore, to understand the roles of RA and oocyte-secreted factors in DNA demethylation in granulosa cells during the follicular development process, granulosa cells were cultured with oocytes, GDF9 and/or RA in the presence of FSH+T. The expression levels of *Dnmt1* and *Tet2* were examined (Figure 6). Furthermore, granulosa cells were cultured with or without FSH+T and/or RA in the absence of fetal bovine serum because serum contains RA precursor retinol. The expression of the genes *Ccnd2*, *Dnmt1*, *Tet2*, *Stk36*, *Trnau1ap* and *Lhcgr* was examined (Figure 7A). Finally, granulosa cells were cultured with FSH+T and/or RA in the absence of fetal bovine serum or cultured with FSH+T and/or 4MP (an ADH inhibitor that suppresses the conversion from retinol to RA) in the presence of serum. Granulosa cells were used for FACS analysis to detect the levels of DNMT1 and DNA in each granulosa cell line (Figure 6B).

Supplementary Table 1. The annotation analysis in MeDIP-seq analysis of demethylated genes in proliferative granulosa cells were done to show the predicted functions in biological process

Genes that are significantly demethylated ($p<0.05$)	p-value
phosphate metabolic process	2.40E-14
phosphorus metabolic process	2.40E-14
phosphorylation	1.50E-11
protein amino acid phosphorylation	4.90E-10
intracellular signaling cascade	6.90E-08
regulation of small GTPase mediated signal transduction	5.40E-07
cytoskeleton organization	1.30E-06
transmembrane transport	1.90E-06
muscle contraction	2.60E-06
protein kinase cascade	4.00E-06
chromatin modification	4.00E-06
muscle system process	8.90E-06
actin cytoskeleton organization	2.60E-05
DNA metabolic process	4.40E-05
cellular macromolecule catabolic process	5.00E-05
lipid catabolic process	5.30E-05
regulation of Ras protein signal transduction	5.30E-05
synapse organization	6.90E-05
actin filament-based process	7.20E-05
macromolecule catabolic process	7.30E-05

Supplementary Table 2. The annotation analysis in MeDIP-seq analysis of demethylated genes in proliferative granulosa cells were done to show the predicted functions in molecular level

Genes that are significantly demethylated ($p<0.05$)	p-value
regulation of transcription	2.50E-05
cytoskeleton organization	6.50E-05
actin cytoskeleton organization	1.20E-04
transcription	1.20E-04
actin filament-based process	2.30E-04
pallium development	5.20E-04
protein amino acid autophosphorylation	5.60E-04
limbic system development	8.50E-04
positive regulation of nucleobase, nucleoside, nucleotide and nucleic acid metabolic process	9.80E-04
telencephalon development	1.10E-03
covalent chromatin modification	1.20E-03
chromatin modification	1.40E-03
regulation of transcription from RNA polymerase II promoter	1.50E-03
chromatin organization	1.70E-03
positive regulation of nitrogen compound metabolic process	1.90E-03
positive regulation of RNA metabolic process	2.00E-03
histone modification	2.20E-03
positive regulation of macromolecule metabolic process	2.20E-03
positive regulation of transcription, DNA-dependent	2.20E-03
negative regulation of macromolecule biosynthetic process	2.70E-03

Supplementary Table 3. Value from MeDIP-seq analysis of the top 9 significantly demethylated genes in granulosa cells

Gene	The predicted gene function	The temporal changes in DNA methylation		
		SF	AF (eCG 0h)	PF (eCG 48h)
Stk36	Serine/threonine kinase	518.2±7.6	0.0±0.0 *	0.0±0.0*
Lnp	ER Junction Formation Factor	574.8±3.7	116.3±116.3*	0.0±0.0*
Selm	Selenoprotein	323.4±4.7	71.1±71.1 *	0.0±0.0*
Tcte3	Organelle biogenesis protein	505.6±1.0	115.4±115.4*	0.0±0.0*
Nfyc	Transcription factor	568.6±4.0	153.9±153.9*	0.0±0.0*
Tead4	Transcription factor	451.0±6.0	145.2±145.2	0.0±0.0*
Spata33	Phosphorylated protein	387.5±2.2	169.4±101.1	0.0±0.0*
Trnau1ap	Nucleotide binding factor	555.5±6.3	303.0±154.0	0.0±0.0*
Lce3c	Antimicrobial factor	444.1±0.9	450.3±45.8 *	0.0±0.0*

Values are represented as the mean ± SEM of three replicates.

＊：$p<0.05$

Supplementary Table 4. Value from MeDIP-seq analysis of imprinted genes in granulosa cells

Gene	Allele	The temporal changes in DNA methylation		
		SF	AF (eCG 0h)	PF (eCG 48h)
Nnat	Paternal	615.16±18.26	603.56±15.60	651.89±3.60
Peg10	Paternal	541.20±12.03	562.40±27.73	548.72±12.81
Pdk4	Maternal	485.29±10.26	393.54±33.94	401.34±13.86
Mest	Paternal	619.11±6.45	572.83±47.00	621.89±12.54
Zim1	Maternal	550.54±17.93	500.93±60.82	468.24±39.36
Peg3	Paternal	425.85±25.90	377.13±54.93	430.60±20.15
Slc22a3	Maternal	474.00±12.27	408.63±27.67	418.43±4.79
Slc22a1	Maternal	455.72±7.59	374.89±40.56	379.58 ± 18.56
Meg3	Maternal	423.86±6.44	347.70±42.11	349.14±25.84
Rian	Maternal	538.67±36.98	421.57±55.10	478.03±27.19

Values are represented as the mean ± SEM of three replicates.

Supplementary Table 5. Primer list for Real-time PCR analysis

Gene	Forward Primer	Reverse Primer	Size (bp)
Kdm6a	5’-GGTTCAAGCTATTGGCTGGT-3’	5’-CATTGCTTCAGCGTTCTCAG-3’	228
Kdm6b	5’-AAGTTCTGCCTCCTGCAGTC-3’	5’-AGTGTGAAGGCATCGTAGGC-3’	299
Dnmt1	5’-GTGGCAAGAAGAAAGGCAAG-3’	5’-GCCCCCTTGTGAATAATCCT-3’	233
Dnmt3a	5’-CCCTTCTTCTGGGCTCTTTGA-3’	5’-CTGTTCATGCCAGGAAGGTT-3’	161
Dnmt3l	5’-CCCTAGGCAGCTCTTGTGAT-3’	5’-CTGAAGGAAGCGGGTAGTTG-3’	170
Uhrf1	5’-CGAGATGACACAGAGCCTGA-3’	5’-GGACTTCTGCTTGCTTTTGC-3’	192
Tet1	5’-CTTCTCCTTTGGCTGTTCGT-3’	5’-CAAGTCGACAGTCTCCAGCA-3’	326
Tet2	5’-CGCACAGTCAGTGAACCATC-3’	5’-CTTCCTGCTCGTTGAGAACC-3’	262
Tet3	5’-ACCCCTACAGCATGAGCAGT-3’	5’-CATGGAGGTCTGGCTTCTTC-3’	219
Ccnd2	5’-TCACCACGTGTTCCCAAGAC-3’	5’-AGCGAATTCCCTCCATCAGA-3’	243
Lhcgr	5’-ACTGGTGTGGTTTCAGGAATT-3’	5’-CCTAAGGAAGGCATAGCCCAT-3’	244
Stk36	5’-GTCCCTGCTGACACACACAG-3’	5’-TAGCAGCAGAAGGTTGAGCA-3’	232
Trnau1ap	5’-ACCCAACAGGCGTGTCTAAG-3’	5’-TAGCCCCACTGGGCATAGTA-3’	235
Spata33	5’-GTCGAAAAGCAAACCCAGAG-3’	5’-CGGGTGATGTTGATGATCTG-3’	249
Lnp	5’-ACCGCCAGAAAGAACTGTTG-3’	5’-ATGAGGGCGTACCTGTTCTG-3’	192
Tcte3	5’-GTGGAACCTCCGTTTGATGA-3’	5’-GTGACAAATGAGGGGCTTTG-3’	202
Lce3c	5’-AGCCTCTTCCTGCTGTGCTA-3’	5’-GGATGCTGATTCCTCCAGAC-3’	237
Nfyc	5’-AAGGTCAGCAGGTGCAGATT-3’	5’-GTGTGATCTGTTGGGCATTG-3’	246
Selm	5’-GAATCCCACTCAGCCAAATG-3’	5’-AGGTCGTCGTGTTCTGAAGC-3’	154
Tead4	5’-TCTGGGCAGACCTCAATACC-3’	5’-TCTCATAGCGGGCATACTCC-3’	168
Ptgs2	5’-TGTACAAGCAGTGGCAAAGG-3’	5’-GCTGTGGATCTTGCACATTG-3’	431
Cyp11a1	5’-GGGAGACATGGCCAAGATGG-3’	5’-CAGCCAAAGCCCAAGTACCG-3’	279
Star	5’-GCAGCAGGCAACCTGGTG-3’	5’-TGATTGTCTTCGGCAGCC-3’	247
Areg	5’-TTACTTTGGCGAACGGTGTG-3’	5’-TGTGCAGTCCCGTTTTCTTG-3’	231
Ereg	5’-ACACTGGTCTGCGATGTGAG-3’	5’-TCCGTAACTTGATGGCACTG-3’	239
Btc	5’-CACAGCACGGTTGATGGACT-3’	5’-CCGAGAGAAGTGGGTTTTCG-3’	217
L19	5’-GGCATAGGGAAGAGGAAGG-3’	5’-GGATGTGCTCCATGAGGATGC-3’	199

Supplementary Table 6. Primer list for Bisulfite sequence, Chip assay, FAIRE-qPCR analysis

Gene	Forward Primer	Reverse Primer	Size (bp)
Stk36 (promoter)	5'-GAGGGGTAGGCCCGTAGTAG-3'	5'-GGGAACTAGCAACAGCTTCG-3'	214
Stk36 (Bisulfite-seq)	5'-GAAAGGATGTTTTTTTTATTTTT-3'	5'-TTTTAACTAACTACCTAAATCTAATACC-3'	112
Trnau1ap (promoter)	5'-GTCCCTCGGAAGCTTATCCT-3'	5'-GACTGCCCCGCTCTTTAATA-3'	151
Trnau1ap (Bisulfite-seq)	5'-ATGTAGGGTAAGAGGATTATTGTG-3'	5'-TCTATAAAAAATACCAAACATAAAAAC-3'	130
Lhcgr (promoter)	5'-AAGTCTTAGGGAAACAGCAATGG-3'	5'-ACCAGCAGCTGTCTCAGAGC-3'	245
Lhcgr (Bisulfite-seq)	5'-GTGAGAGGGGAGGGTTGGAG-3'	5'-TTCAAGACCAACATTACCAACACCA-3'	199
Spata33 (promoter)	5'-GGTCCTAGTCGGAGGTCTCG-3'	5'-GACGGTAAGGGGCTGTCAT-3'	201
Lnp (promoter)	5'-CCCACTCTTCTCTCGCTCAG-3'	5'-GGTGTGGCAGGAGGATTTAG-3'	202
Tcte3 (promoter)	5'-CCCCCTCTCATCAAACACAG-3'	5'-TGCGTCCCATCCCTTATTCT-3'	145
Lce3c (promoter)	5'-AGAAGGGTCGTCCATTCAAG-3'	5'-TGAGAGCAGACACACCCAAA-3'	288
Nfyc (promoter)	5'-CGCATCCTAGCTGAGAGCTT-3'	5'-GGCTGAGGTGCCTGAAGTC-3'	161
Selm (promoter)	5'-TCTCCTAGGTTCCTGCTTCC-3'	5'-ACTAACCCCCATCCCTGTCT-3'	222
Tead4 (promoter)	5'-TGTGAATGGGATGTGACCAG-3'	5'-GACCGAGAGAGGCGATCTAA-3'	178
Ptgs2 (promoter)	5'-AGGGGAAGCTGTGACACTCT-3'	5'-CACGCAAATGAGAGACGAAG-3'	208
Cyp11a1 (promoter)	5'-ACTGAGGCAGGGGGATCTTA-3'	5'-GCGTTGGTGTTTGACCTACA-3'	244
Star (promoter)	5'-CTGGGTGGGTCATCTCATTT-3'	5'-TCCATCAGGTCTGGAGGGTA-3'	236
Areg (promoter)	5'-GTCAGCTCCTCAGCACCACT-3'	5'-CGAGCACCTGCTGCTTTTAT-3'	197
Ereg (promoter)	5'-TGAAACCCTCCTCTCAGGTG-3'	5'-CAGCGCAGCGACCTTTAT-3'	168
Btc (promoter)	5'-ATGACCAGGTCCTCTTCTGC-3'	5'-AGCTGCCCCACACCCATA-3'	166

Supplementary Table 7. The antibody lists

Target	Available	The name of antibody	Catalog No.	Species	Experiment
DNMT1	WB, IHC, IP	anti-DNMT1 antibody [APC]	Novus Biologicals (NB100-56519APC)	mouse	Flow cytometry
DNMT1	WB, IHC, IF, IP, Chip assay	anti-DNMT1 polyclonal antibody	GeneTex (GTX116011)	rabbit	WB, IF
PCNA	WB, IHC, IF, IP, FC	anti-PCNA (PC10) antibody	Cell signaling (2586S)	mouse	IF
H3K27ac	WB, IF, FC, IP, Chip assay, CR, E	anti-acetyl-Histone H3(K27) antibody	Cell signaling (8173S)	rabbit	WB, Chip assay
TET2	WB, IF, IP	anti-TET2 polyclonal antibody	Abcam (ab124297)	rabbit	IF
TET2	WB, IP, IF, F	anti-TET2 (D6C7K) antibody	Cell signaling (36449)	rabbit	WB
β-actin	WB, IHC, IF, E	anti-β actin monoclonal antibody	Sigma (A5316)	mouse	WB

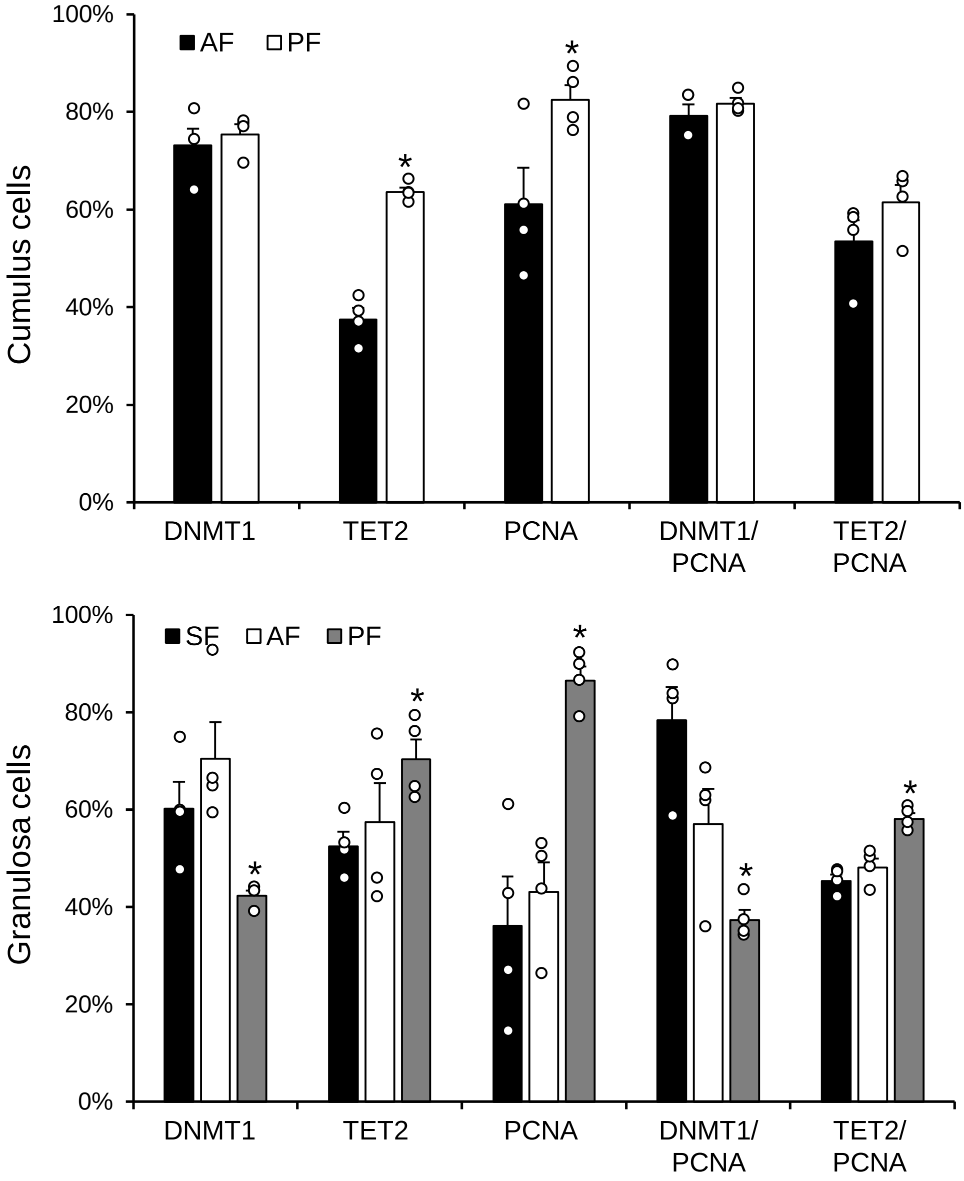

Supplementary Figure 1. The percentage of DNMT1-positive cells/total number of cells, TET2-positive cells/total number of cells, PCNA-positive cells/total number of cells, DNMT1-positive cells/PCNA-positive cells and TET2-positive cells/PCNA-positive cells in cumulus cells or granulosa cells is shown in Figure 1c.

SF; Secondary follicle with multilayered granulosa cells. AF; Antral follicle (3-week mice not treated with eCG). PF; Preovulatory follicle (at 48 h after eCG injection). *; Significant differences were observed compared to those of SF or AF ($p<0.05$). Significant differences in percentage values were transformed into normally distributed numbers by angle transformation and then analyzed by one-way ANOVA. Tukey–Kramer was used as post hoc test. Experiments are represented as means±SEM. n=4 biological replicates.

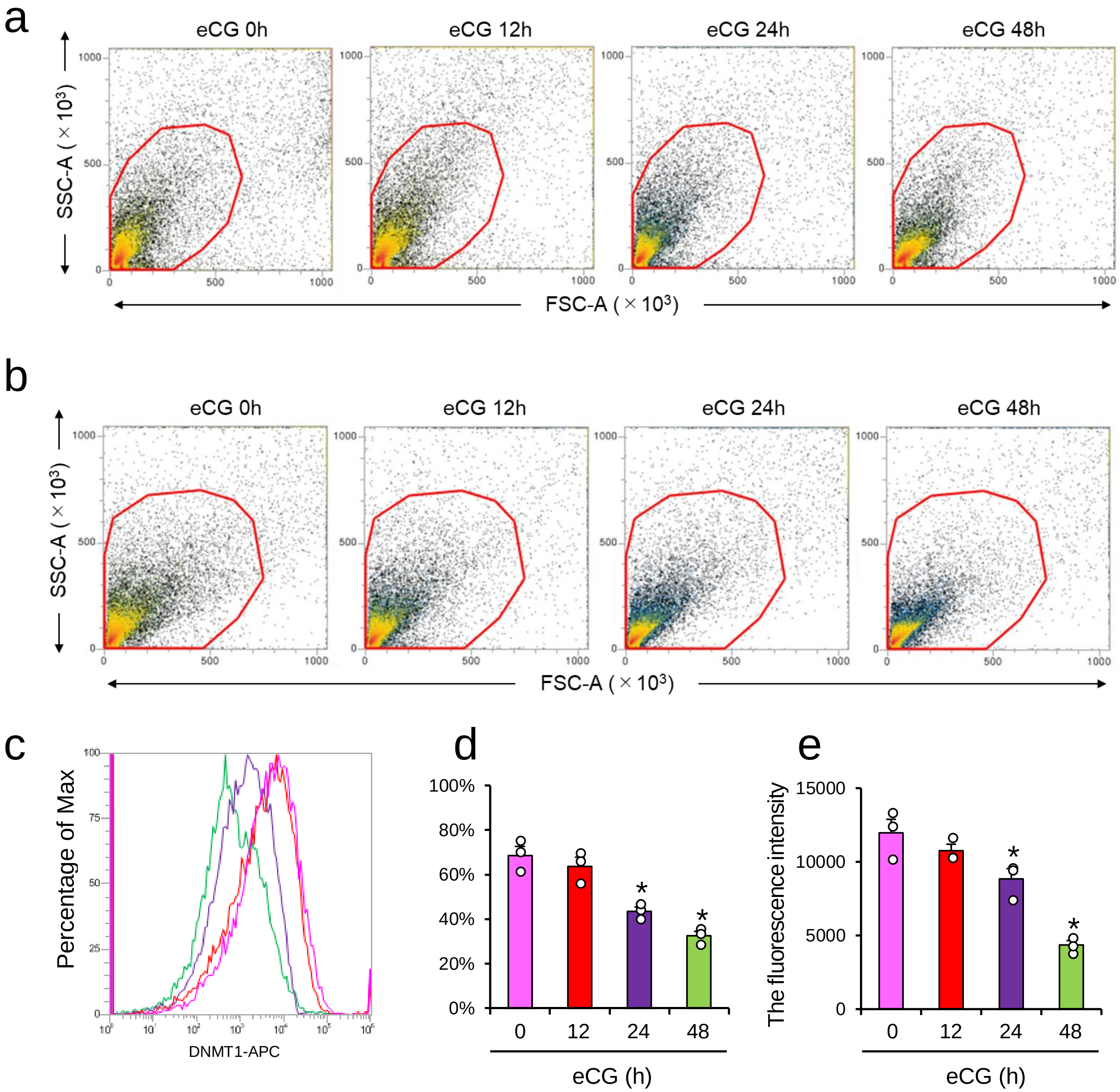

Supplementary Figure 2. Gating and the percentage of DNMT1 in proliferative granulosa cells in flow cytometry analysis

a: To identify PI-positive mouse granulosa cells in Figure 2a, a red gate was made, and 20,000 granulosa cells surrounded by a red line were analyzed. Each histogram was drawn, and the percentage was calculated.
b: To identify DNMT1-positive mouse granulosa cells in Figure 2b, a red gate was made, and 20,000 granulosa cells surrounded by a red line were analyzed. Each histogram was drawn, and the percentage was calculated.
c,d,e: (c) Histogram of DNMT1 fluorescence in granulosa cells during follicular development. (d) The percentage of DNMT1 in granulosa cells during follicular development. (e) The mean fluorescence intensity was analyzed in DNMT1-positive stained granulosa cells. *; Significant differences were observed compared to those of eCG at 0 hours ($p<0.05$). Significant differences in percentage values were transformed into normally distributed numbers by angle transformation and then analyzed by one-way ANOVA. Tukey–Kramer was used as post hoc test. Experiments are represented as means±SEM. n=3 biological replicates.

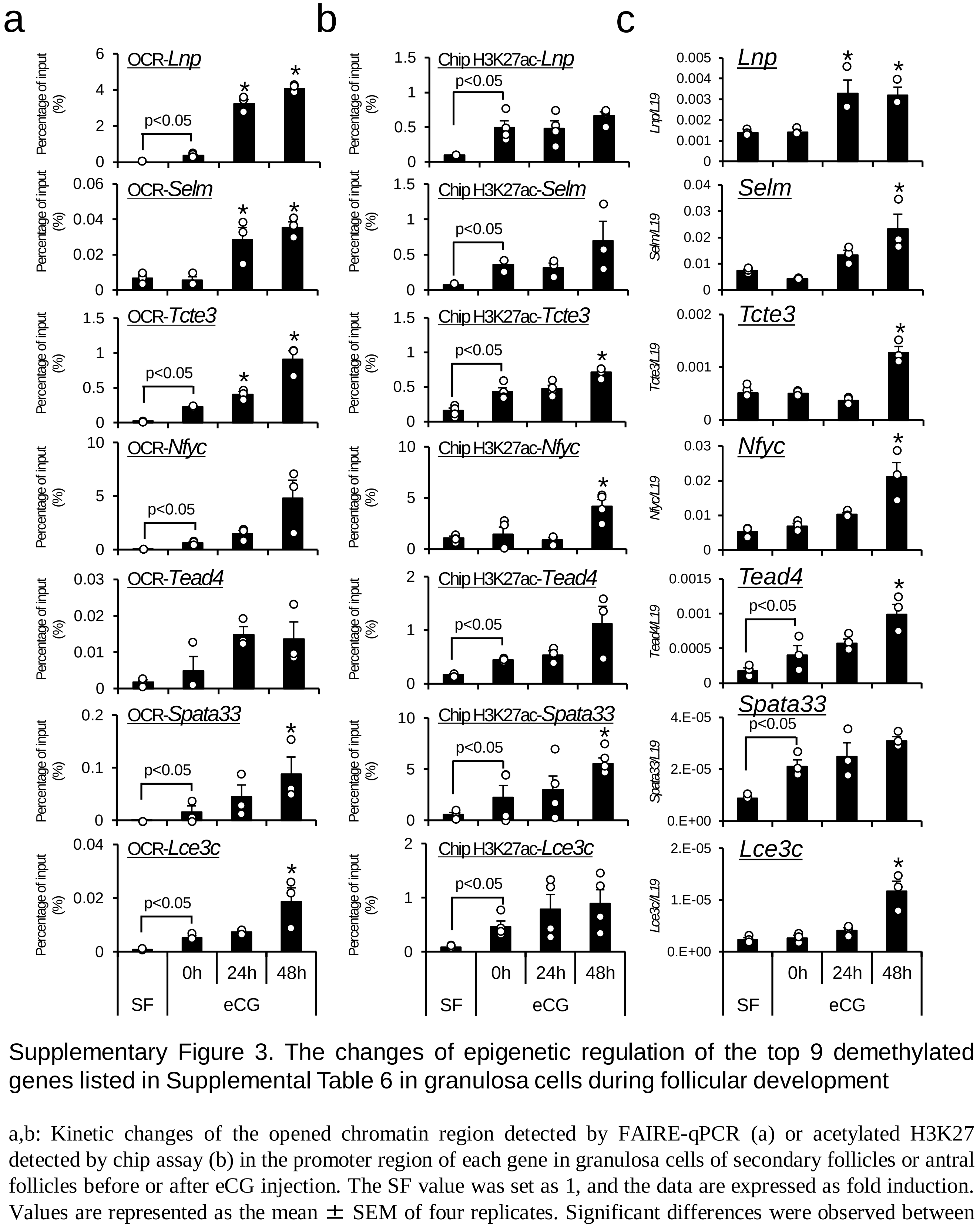

Supplementary Figure 3. The changes of epigenetic regulation of the top 9 demethylated genes listed in Supplemental Table 6 in granulosa cells during follicular development

a,b: Kinetic changes of the opened chromatin region detected by FAIRE-qPCR (a) or acetylated H3K27 detected by chip assay (b) in the promoter region of each gene in granulosa cells of secondary follicles or antral follicles before or after eCG injection. The SF value was set as 1, and the data are expressed as fold induction. Values are represented as the mean ± SEM of four replicates. Significant differences were observed between SF and eCG at 0 hours ($p<0.05$). *; Significant differences were observed between eCG at 0 hours and eCG-primed mice ($p<0.05$). SF; secondary follicles

c: Kinetic changes of the expression of each gene in granulosa cells. Levels of mRNA were normalized to that of *L19*. Values are represented as the mean ± SEM of three replicates. Significant differences were observed between SF and eCG at 0 hours ($p<0.05$). *; Significant differences were observed between eCG at 0 hours and eCG-primed mice ($p<0.05$). SF; secondary follicles.

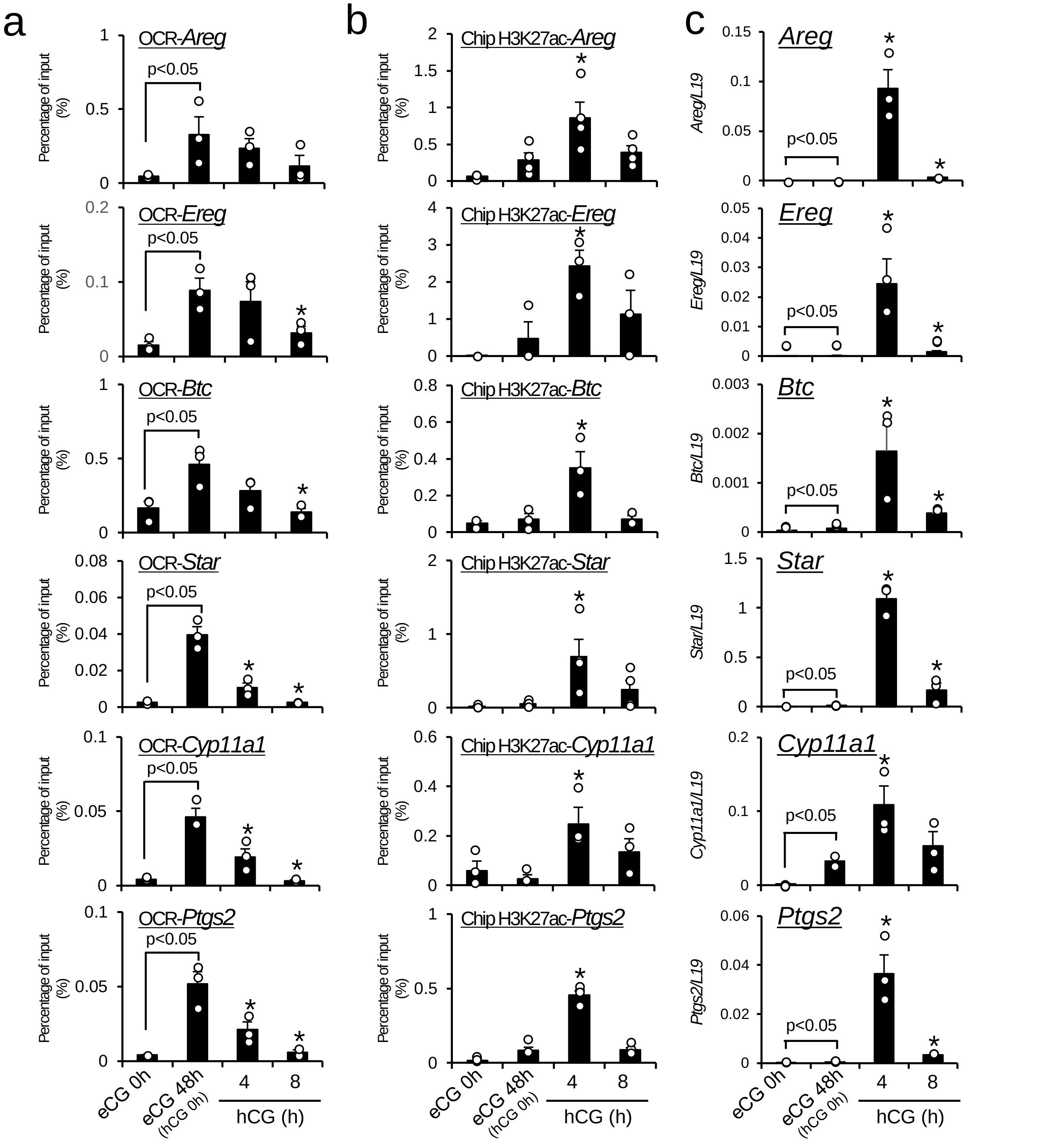

Supplementary Figure 4. Epigenetic regulation in promoter regions of LH-targeting genes in granulosa cells

a,b: Kinetic changes of the opened chromatin region detected by FAIRE-qPCR (a) or acetylated H3K27ac detected by chip assay (b) in the promoter region of each gene in granulosa cells before and after hCG injection. The eCG 0 h value was set as 1, and the data are expressed as fold induction. Values are represented as the mean ± SEM of three replicates. hCG 0 h=eCG 48 h. Significant differences were observed between eCG 0 h and eCG 48 h ($p<0.05$). *; Significant differences were observed compared to those of hCG 0 h (eCG 48 h) ($p<0.05$).
c: Kinetic changes of the expression of each gene in granulosa cells before or after hCG injection. Levels of mRNA were normalized to that of L19. Values are mean ± SEM of three replicates. hCG 0 h=eCG 48 h. Significant differences were observed between eCG 0 h and eCG 48 h ($p<0.05$). *; Significant differences were observed compared to those of hCG 0 h (eCG 48 h) ($p<0.05$).

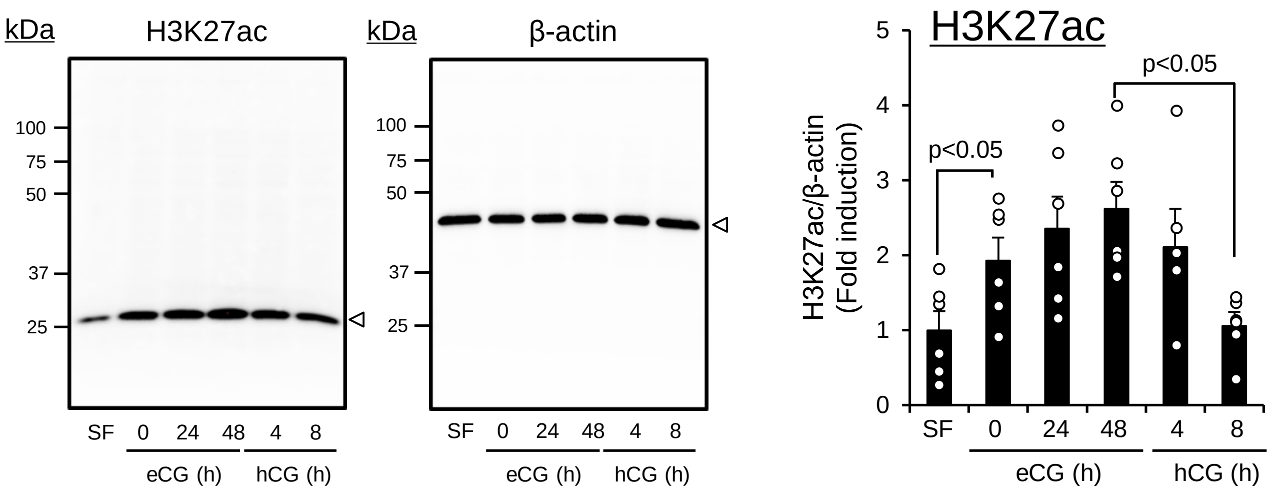

Supplementary Figure 5. Temporal changes of H3K27ac in granulosa cells during follicular development and the ovulation process.

Quantitative expression of H3K27ac relative to the expression of β-actin (a loading control), as determined by western blotting. The results are representative of three independent experiments. The SF value was set as 1, and the data are expressed as fold induction. The values are the mean ± SEM of six replicates. Significant differences were observed between SF and eCG 0 h or between hCG 0 h (eCG 48 h) and hCG 8 h ($p<0.05$). *; Significant differences were observed compared to those of eCG 0 h ($p<0.05$). SF; Secondary follicles.

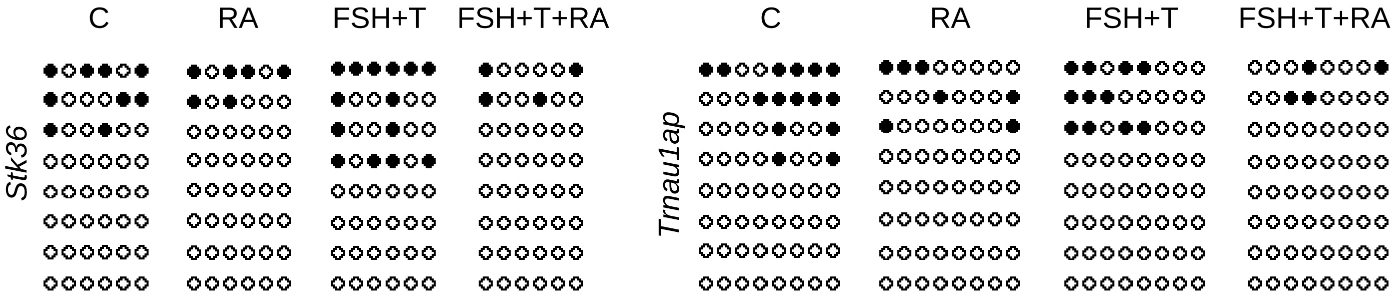

● methylated cytosine
○ demethylated cytosine

	C	RA	FSH+T	FSH+T+RA
Stk36 (%)	26.5±5.0	18.3±4.8	21.0±8.9	9.1±1.6 *
Trnau1ap (%)	24.7±4.3	13.3±3.0	16.2±1.1	6.5±2.0 *

*; $p<0.05$

Supplementary Figure 6. The effects of RA on the temporal changes of cytosine methylation in each promoter region of Stk36 and Trnau1ap in granulosa cells cultured with or without FSH and testosterone (FSH+T).

Granulosa cells were collected from ovaries of 3-week-old mice after treatment with eCG for 6 h and treated with RA and/or FSH plus testosterone (FSH+T) in the absence of serum. ●; Methylated cytosine. ○; Unmethylated cytosine. *; Significant differences were observed between the control group and the RA treatment group in the presence of FSH+T ($p<0.05$). n=3 biological replicate. Significant differences in percentage values were transformed into normally distributed numbers by angle transformation and then analyzed by one-way ANOVA. Tukey–Kramer was used as post hoc test.

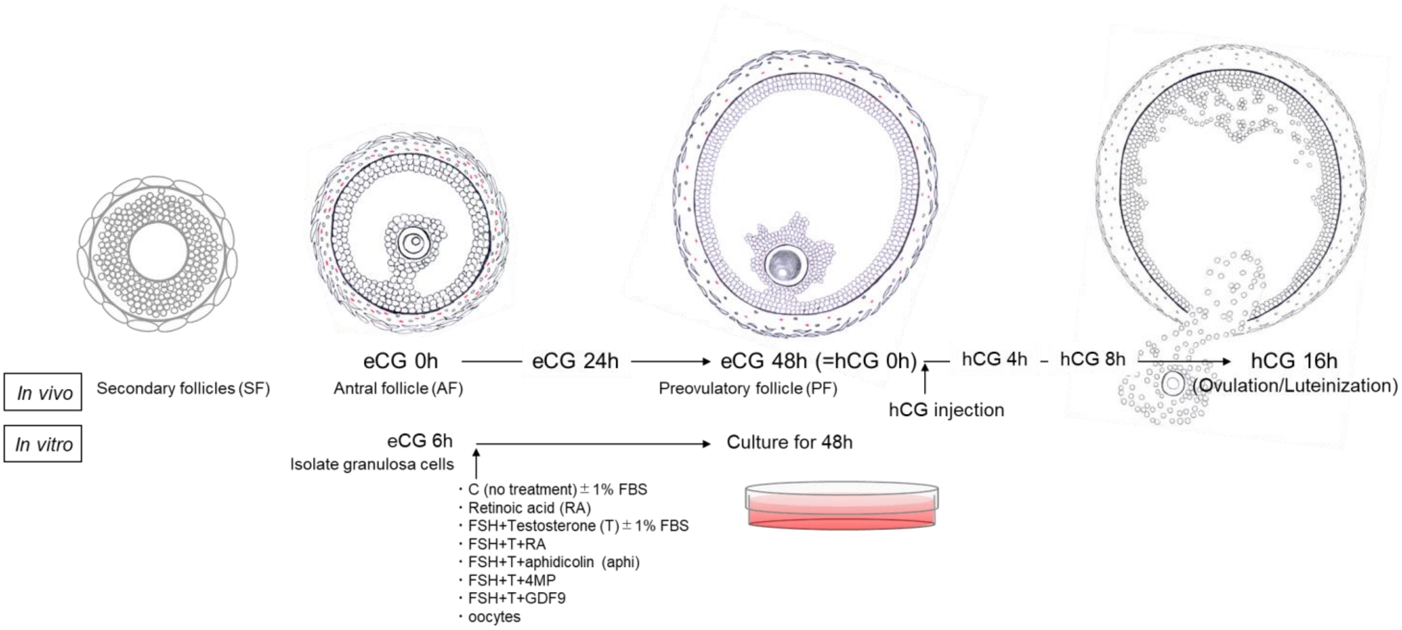

Supplementary Figure 7. Time point of sample collection when granulosa cells were collected in *in vivo* and *in vitro* experiments.

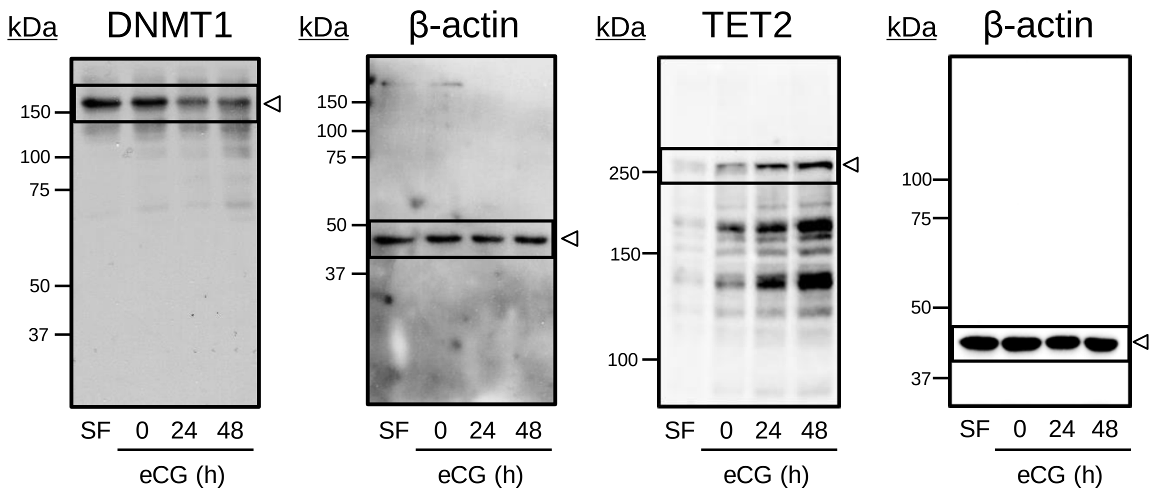

Supplementary Figure 8. Full-length blotting images of westernblotting

Full-length blotting images of DNMT1, TET2 and β-actin in granulosa cells of secondary follicles with multilayered granulosa cells or in eCG-stimulated granulosa cells in Figure 1b,c.
