# Supplementary material for: Large-scale DNA demethylation occurs in proliferating ovarian granulosa cells during mouse follicular development
Source: Commun Biol. 2021 Nov 25;4:1334. doi: 10.1038/s42003-021-02849-w (PMC8617273; doi:10.1038/s42003-021-02849-w)
Supplement: Supplementary file 3 — Description of Additional Supplementary Files [file 42003_2021_2849_MOESM3_ESM.pdf]

Description of Additional Supplementary Files

File name: Supplementary Data 1.
Description: Low data in Figures and supplementary figures.
