# Supplementary material for: Large-scale DNA demethylation occurs in proliferating ovarian granulosa cells during mouse follicular development
Source: Commun Biol. 2021 Nov 25;4:1334. doi: 10.1038/s42003-021-02849-w (PMC8617273; doi:10.1038/s42003-021-02849-w)
Supplement: Supplementary file 5 — Reporting Summary [file 42003_2021_2849_MOESM5_ESM.pdf]

nature research

Corresponding author(s): Masayuki Shimada, PhD

Last updated by author(s): Oct 27, 2021

Reporting Summary

Nature Research wishes to improve the reproducibility of the work that we publish. This form provides structure for consistency and transparency in reporting. For further information on Nature Research policies, see our Editorial Policies and the Editorial Policy Checklist.

Statistics

For all statistical analyses, confirm that the following items are present in the figure legend, table legend, main text, or Methods section.

n/a	Confirmed	
☐	☒	The exact sample size (n) for each experimental group/condition, given as a discrete number and unit of measurement
☐	☒	A statement on whether measurements were taken from distinct samples or whether the same sample was measured repeatedly
☐	☒	The statistical test(s) used AND whether they are one- or two-sided<br>*Only common tests should be described solely by name; describe more complex techniques in the Methods section.*
☐	☒	A description of all covariates tested
☒	☐	A description of any assumptions or corrections, such as tests of normality and adjustment for multiple comparisons
☐	☒	A full description of the statistical parameters including central tendency (e.g. means) or other basic estimates (e.g. regression coefficient) AND variation (e.g. standard deviation) or associated estimates of uncertainty (e.g. confidence intervals)
☐	☒	For null hypothesis testing, the test statistic (e.g. F, t, r) with confidence intervals, effect sizes, degrees of freedom and P value noted<br>*Give P values as exact values whenever suitable.*
☒	☐	For Bayesian analysis, information on the choice of priors and Markov chain Monte Carlo settings
☒	☐	For hierarchical and complex designs, identification of the appropriate level for tests and full reporting of outcomes
☒	☐	Estimates of effect sizes (e.g. Cohen's d, Pearson's r), indicating how they were calculated

Our web collection on statistics for biologists contains articles on many of the points above.

Software and code

Policy information about availability of computer code

Data collection	No software were used.
Data analysis	In MeDIP-Seq analysis, the methylated region was identified by MEDIPS software (http://www.bioconductor.org/packages/2.13/bioc/html/MEDIPS.html). (Bioconductor version: 2.13, Version: 1.12.0, Boston, USA).<br><br>The prediction of gene function was analyzed using Functional Annotation Bioinformatics Microarray Analysis (https://david-d.ncifcrf.gov/tools.jsp). (DAVID Bioinformatics Resources 6.7, MD, USA).<br><br>The percentage of methylation of CpG sites in promoter region was analyzed by QUantification tool for Methylation Analysis (http://quma.cdb.riken.jp/).<br><br>Cluster analysis and heat map analysis of promoter region was done using heatmap.2 from gplots in the R package of Software R (Version 3.6.1) (https://www.r-project.org/).

For manuscripts utilizing custom algorithms or software that are central to the research but not yet described in published literature, software must be made available to editors and reviewers. We strongly encourage code deposition in a community repository (e.g. GitHub). See the Nature Research guidelines for submitting code & software for further information.

Data

Policy information about availability of data

All manuscripts must include a data availability statement. This statement should provide the following information, where applicable:

- Accession codes, unique identifiers, or web links for publicly available datasets
- A list of figures that have associated raw data
- A description of any restrictions on data availability

Raw data and processed Chip-seq (MeDIP-Seq) data from our study can be accessed via DNA Data Bank of Japan (DDBJ) web site. The accession number of raw data is DRA010809 (http://trace.ddbj.nig.ac.jp/dra/index_e.html). The accession number of final processed data is E-GEAD-400.

Field-specific reporting

Please select the one below that is the best fit for your research. If you are not sure, read the appropriate sections before making your selection.

☒ Life sciences ☐ Behavioural & social sciences ☐ Ecological, evolutionary & environmental sciences

For a reference copy of the document with all sections, see nature.com/documents/nr-reporting-summary-flat.pdf

Life sciences study design

All studies must disclose on these points even when the disclosure is negative.

Sample size	n=3 or n>3 samples for each treatment group in each analysis.
Data exclusions	No data were excluded from all analyses.
Replication	All attempts at replication were successful.
Randomization	Not randomised.
Blinding	There was no blinding in this study.

Reporting for specific materials, systems and methods

We require information from authors about some types of materials, experimental systems and methods used in many studies. Here, indicate whether each material, system or method listed is relevant to your study. If you are not sure if a list item applies to your research, read the appropriate section before selecting a response.

Materials & experimental systems

n/a	Involved in the study	
☐	☒	Antibodies
☒	☐	Eukaryotic cell lines
☒	☐	Palaeontology and archaeology
☐	☒	Animals and other organisms
☒	☐	Human research participants
☒	☐	Clinical data
☒	☐	Dual use research of concern

Methods

n/a	Involved in the study	
☐	☒	ChIP-seq
☐	☒	Flow cytometry
☒	☐	MRI-based neuroimaging

Antibodies

Antibodies used

Information on the antibodies used in this paper is shown in Supplemental table 7.
IF (immunofluorescence);
Primary antibody: Anti-PCNA (PC10) antibody (Cell signaling: 2586S, MA, USA), Anti-DNMT1 polyclonal antibody (GeneTex: GTX116011, LA, USA), Anti-TET2 polyclonal antibody (Abcam: ab124297, Cambridge, UK),
Secondary antibody: FITC-conjugated goat anti-mouse IgG secondary antibody (Sigma), Alexa Fluor 568 goat anti-rabbit secondary antibody (Sigma),

WB (Western blotting analysis);
Primary antibody: Anti-Acetyl-Histone H3(K27) antibody (Cell signaling: 8173S), Anti-DNMT1 polyclonal antibody (GeneTex: GTX116011), Anti-TET2 (D6C7K) antibody (Cell signaling: 36449), Anti-β actin monoclonal antibody (Sigma: A5316)
Secondary antibody: Anti-rabbit IgG, HRP-linked Antibody (Cell signaling), Anti-mouse IgG, HRP-linked Antibody (Cell signaling).

FC (Flow cytometric analysis);

	Anti-DNMT1 antibody conjugated APC (Novus Biologicals: NB100-56519APC, Colorado, USA).
Validation	Anti-PCNA (PC10) antibody (Cell signaling: 2586S) is available for Western blotting analysis (WB), Immunohistochemistry (IHC), Immunofluoresence (IF), Immunoprecipitation (IP) and Flow cytometric analysis (FC). The reactivity is human, mice, rat, monkey, bovine and pig. This information is shown in https://www.cellsignal.jp/products/primary-antibodies/pcna-pc10-mouse-mab/2586?Ntk=Products&Ntt=2586.<br><br>Anti-DNMT1 polyclonal antibody (GeneTex: GTX116011) is available for Western blotting analysis (WB), Immunohistochemistry (IHC), Immunofluoresence (IF), Immunoprecipitation (IP) and Chip assay. The reactivity is human. This information is shown in https://www.genetex.com/Product/Detail/DNMT1-antibody-N1-N-term/GTX116011.<br><br>Anti-TET2 polyclonal antibody (Abcam: ab124297) is available for Western blotting analysis (WB), Immunofluoresence (IF) and Immunoprecipitation (IP). The reactivity is mouse. This information is shown in https://www.abcam.co.jp/tet2-antibody-ab124297.html.<br><br>Anti-TET2 (D6C7K) antibody (Cell signaling: 36449) is available for Western blotting analysis (WB), Immunofluoresence (IF), Immunoprecipitation (IP) and Flow cytometric analysis (F). The reactivity is mice. This information is shown in https://www.cellsignal.jp/products/primary-antibodies/tet2-d6c7k-rabbit-mab-mouse-specific/36449.<br><br>Anti-acetyl-Histone H3(K27) antibody (Cell signaling: 8173S) is available for Western blotting analysis (WB), Immunofluoresence (IF), Flow cytometric analysis (FC), Immunoprecipitation (IP), Chip assay, CUT&RUN Assay and ELISA. The reactivity is human, mice, rat and monkey. This information is shown in https://www.cellsignal.jp/products/primary-antibodies/acetyl-histone-h3-lys27-d5e4-xp-rabbit-mab/8173?Ntk=Products&Ntt=8173.<br><br>Anti-β actin monoclonal antibody (Sigma: A5316) is available for Western blotting analysis (WB), Immunohistochemistry (IHC), Immunofluoresence (IF) and ELISA. The reactivity is Drosophila, Hirudo medicinalis, carp, rabbit, wide range, pig, cat, human, rat, chicken, guinea pig, sheep, mouse, bovine, canine. This information is shown in https://www.sigmaaldrich.com/catalog/product/sigma/a5316?lang=ja®ion=JP.<br><br>Anti-DNMT1 antibody conjugated APC (Novus Biologicals: NB100-56519APC) is available for Western blotting analysis (WB), Chip assay, Immunohistochemistry (IHC) and Immunoprecipitation (IP). The reactivity is Human, Mouse, Zebrafish. This information is shown in https://fnkprddata.blob.core.windows.net/domestic/data/datasheet/NOV/NB100-56519PCP.pdf.

Animals and other organisms

Policy information about studies involving animals; ARRIVE guidelines recommended for reporting animal research

Laboratory animals	Immature female (3 weeks old) C57BL/6 mice were obtained from Charles River Laboratories Japan.
Wild animals	This study did not involve wild animals.
Field-collected samples	This study did not involve samples collected from the field.
Ethics oversight	The animal study was approved by the Hiroshima University Animal Committee (Permit Number: C18-34), and the mice were maintained in accordance with the Hiroshima University Guidelines for the Care and Use of Laboratory Animals.?

Note that full information on the approval of the study protocol must also be provided in the manuscript.

ChIP-seq

Data deposition

☒ Confirm that both raw and final processed data have been deposited in a public database such as GEO.

☒ Confirm that you have deposited or provided access to graph files (e.g. BED files) for the called peaks.

Data access links<br>*May remain private before publication.*	The DDBJ accession number of the raw data produced in this manuscript is DRA010809 (http://trace.ddbj.nig.ac.jp/dra/index_e.html).<br>The DDBJ accession number of the processed data files produced in this manuscript is DRP006540 (https://ddbj.nig.ac.jp/DRASearch/study?acc=DRP006540).
Files in database submission	-Raw sequencing files (FASTQ Format)<br>SF_1_1.txt, SF_1_2.txt, SF_2_1.txt, SF_2_2.txt, SF_3_1.txt, SF_3_2.txt, AF_1_1.txt, AF_1_2.txt, AF_2_1.txt, AF_2_2.txt, AF_3_1.txt, AF_3_2.txt, PF_1_1.txt, PF_1_2.txt, PF_2_1.txt, PF_2_2.txt, PF_3_1.txt, PF_3_2.txt<br><br>-Processed data files (TXT Format)<br>SF1.ROI_CGI_promoter.final.txt, SF2.ROI_CGI_promoter.final.txt, SF3.ROI_CGI_promoter.final.txt, AF1.ROI_CGI_promoter.final.txt, AF2.ROI_CGI_promoter.final.txt, AF3.ROI_CGI_promoter.final.txt, PF1.ROI_CGI_promoter.final.txt, PF2.ROI_CGI_promoter.final.txt, PF3.ROI_CGI_promoter.final.txt<br><br>SF1.ROI_nonCGI_promoter.final.txt, SF2.ROI_nonCGI_promoter.final.txt, SF3.ROI_nonCGI_promoter.final.txt, AF1.ROI_nonCGI_promoter.final.txt, AF2.ROI_nonCGI_promoter.final.txt, AF3.ROI_nonCGI_promoter.final.txt,

	PF1.ROI_nonCGI_promoter.final.txt, PF2.ROI_nonCGI_promoter.final.txt, PF3.ROI_nonCGI_promoter.final.txt
Genome browser session (e.g. UCSC)	Using UCSC Genome browser on mouse (mm10) assembly (https://genome.ucsc.edu/cgi-bin/hgTracks?db=mm10&lastVirtModeType=default&lastVirtModeExtraState=&virtModeType=default&virtMode=0&nonVirtPosition=&position=chr17%3A88723598%2D88805652&hgsid=928781575_LAwyitF9bAekEfv1V6uiCqNZPezH) or IVG software (Version 2.8.0), we uploaded a bigWig file to DDBJ (accession number; DRP006540, https://ddbj.nig.ac.jp/DRASearch/study?acc=DRP006540). for visualizing the methylation status of the promoter region of each gene. The file names are as follows, BigWig_SF_1.bigwig, BigWig_SF_2.bigwig, BigWig_SF_3.bigwig, BigWig_AF_1.bigwig, BigWig_AF_2.bigwig, BigWig_AF_3.bigwig, BigWig_PF_1.bigwig, BigWig_PF_2.bigwig, BigWig_PF_3.bigwig

Methodology

Replicates	All MEDIP-seq experiments were done in 3 biological duplicates. For each biological replicate at each stage, we pooled DNAs purified from granulosa cells of 4 ovaries (2 female mice) before MEDIP-seq.
Sequencing depth	All sequencing data were done as 300 bp paired-end reads with opposite direction. Sequencing were done to achieve 4.0-7.5 billion reads per biological replicates.
Antibodies	The fragmented DNA was dissociated into single strands, then methylated region was enriched by Methyl-CpG binding domain of human MBD2 protein using MetylMiner Methylated DNA Enrichment Kit (#ME10025, Invitrogen, Manual part #A11129).
Peak calling parameters	MACS2 was used with default settings for peak calling when converting a .bam file to a .bed file by BEDtools (Version 2.21.0) (https://bedtools.readthedocs.io/en/latest/content/history.html). The setting information is contained in raw data files.
Data quality	To check the quality of the sequence data, we used FastQC (Version 0.10.1) (http://www.bioinformatics.babraham.ac.uk/projects/fastqc/).
Software	To analyze the data derived from Methylated DNA Immunoprecipitation (MeDIP) experiments, we used the MEDIPS software package (Version 1.12.0) (http://www.bioconductor.org/packages/2.13/bioc/html/MEDIPS.html).<br>To check the quality of the sequence data, we used FastQC (Version 0.10.1) (http://www.bioinformatics.babraham.ac.uk/projects/fastqc/).<br>The prediction of gene function was analyzed using Functional Annotation Bioinformatics Microarray Analysis (https://david-d.ncifcrf.gov/tools.jsp). (DAVID Bioinformatics Resources 6.7, MD, USA).<br>Cluster analysis and heat map analysis of promoter region was done using heatmap.2 from gplots in the R package of Software R (Version 3.6.1) (https://www.r-project.org/).

Flow Cytometry

Plots

Confirm that:

☒ The axis labels state the marker and fluorochrome used (e.g. CD4-FITC).

☒ The axis scales are clearly visible. Include numbers along axes only for bottom left plot of group (a 'group' is an analysis of identical markers).

☐ All plots are contour plots with outliers or pseudocolor plots.

☒ A numerical value for number of cells or percentage (with statistics) is provided.

Methodology

Sample preparation	For detection of DNMT1, granulosa cells were fixed with 66 % (v/v) methanol in PBS (-) for 10 min at 4 °C. After washing, these cells were treated with 0.1 % (v/v) Triton X-100 in DMEM/F12 at room temperature for 15 min, and then further treated with 5 % (v/v) FBS in DMEM/F12 at room temperature for 30 min. The primary antibody (1:50 anti-DNMT1 antibody, Supplemental table 3) was added at 37 °C for 1 h. For detection of propidium iodide (PI) signal, fixed granulosa cells were treated with RNase A (Qiagen Sciences) and PI staining reagents (Cellstain®-PI solution, P378) at 37 °C for 30 min.
Instrument	Attune NxT Acoustic Focusing Cytometer (Invitrogen)
Software	Attune NxT Acoustic Focusing Cytometer software (Invitrogen)
Cell population abundance	The fluorescent intensity of 20,000 cell-specific events was detected using Attune NxT Acoustic Focusing Cytometer Ver 2.6 (ThermoFisher). No post-sort fractions were collected.
Gating strategy	We applied forward and side scatter parameters (FSC, SSC) to exclude cell debris and doublets as shown in Supplemental Figure 4C, B.

☒ Tick this box to confirm that a figure exemplifying the gating strategy is provided in the Supplementary Information.
